# Impact of Sodium Zirconium Cyclosilicate Therapy on Nutrition Status in Patients with Hyperkalemia

**DOI:** 10.3390/jcm12010083

**Published:** 2022-12-22

**Authors:** Teruhiko Imamura, Hayato Fujioka, Nikhil Narang, Koichiro Kinugawa

**Affiliations:** 1 Second Department of Medicine, University of Toyama, Toyama 930-0194, Japan; 2 Advocate Christ Medical Center, Oak Lawn, IL 60453, USA

**Keywords:** chronic kidney disease, potassium, hyperkalemia

## Abstract

Background: Restriction of oral potassium intake is a necessary dietary intervention for managing chronic hyperkalemia. These dietary changes may have negative impacts on nutrition status, particularly in geriatric cohorts with multiple comorbidities. Sodium zirconium cyclosilicate (SZC) is a newly introduced potassium binder intended for patients with hyperkalemia. We aimed to investigate whether the improvements in hyperkalemia with SZC therapy and the liberation of potassium intake restriction may improve nutrition status in a primarily geriatric patient cohort with chronic hyperkalemia. Methods: Patients who were maintained on SZC therapy for at least 3 months were retrospectively studied. Following the initiation of SZC and improvement in hyperkalemia, instructions on the restriction of potassium intake were loosened according to the institutional protocol. The change in nutrition status during the 3 month therapeutic period using SZC was investigated by referencing the prognostic nutritional index score (PNI), geriatric nutritional risk index score (GNRI), and controlling nutritional status (CONUT) scores. Results: A total of 24 patients (median age 78 years, 58% men, median estimated glomerular filtration rate 29.8 mL/min//1.73 m^2^) were included. Serum potassium level decreased significantly from 5.4 (5.1, 5.9) to 4.4 (4.2, 4.9) mEq/L without any drug-related adverse events, including hypokalemia. Nutrition-related scores, including the PNI score, the GNRI score, and the CONUT score, improved significantly following 3 months of SZC therapy (*p* < 0.05 for all). Psoas muscle volume and average days for one movement also improved significantly during the therapeutic period (*p* < 0.05 for both). Conclusions: Mid-term SZC therapy and liberation of potassium intake restriction might improve nutrition status in geriatric patients with chronic hyperkalemia.

## 1. Introduction

Hyperkalemia is associated with mortality and morbidity, particularly in elderly patients with chronic kidney disease following adjustment for various background confounders [1]. A restriction of potassium intake is often instructed to manage hyperkalemia [2]. However, severe potassium restriction may potentially contribute to the progression of malnutrition and sarcopenia in elderly patients with multiple comorbidities dominantly due to reduced consumption of other essential nutrition [3].

Sodium zirconium cyclosilicate (SZC) is a recently introduced non-polymer zirconium silicate compound that decreases serum potassium levels by exchanging sodium and hydrogen for potassium and ammonium ions in the gastrointestinal tract [4]. Given its robust evidence and few drug-related adverse events, its use has increased thus far as compared with other conventional potassium binders. 

We hypothesized that the use of SZC may allow for more liberalization of potassium intake and therefore improve nutrition status without recurrent hyperkalemia in patients with chronic hyperkalemia who receive strict restriction of potassium intake beforehand. In this proof-of-concept retrospective study, we investigated the change in nutritional status following SZC initiation and liberalization of potassium intake in patients with chronic hyperkalemia. 

## 2. Methods

### 2.1. Patient Selection

All patients who received SZC for over 3 months between July 2020 and October 2022 to treat hyperkalemia (defined as a serum potassium level >5.0 mEq/L) were retrospectively included. In general, all patients were seen at the outpatient clinic every two weeks for laboratory data measurements. Body weight gain, physical examination including peripheral edema, and pulmonary congestion in chest X-ray were also followed at an out-patient clinic. Patients taking sodium polystyrene sulfonate were excluded. Patients who terminated SZC within 3 months were excluded. Informed consent was obtained from all participants, and the present study was approved by the institutional ethical board beforehand. Data were retrieved by electrical chart review.

### 2.2. Study Design

All patients were followed from the initiation of SZC (day 0) to the 3 month follow-up. If patients died during the 3 month observational period, follow-up data was collected before the time of death. The primary outcome was a change in the calculated nutrition scores, as detailed below, from baseline to the 3 month follow-up. 

### 2.3. SZC Therapy

SZC was initiated to treat hyperkalemia at a loading dose of 30 g/day for 2 days, in principle, followed by a maintenance dose of 5–15 g/day according to the levels of serum potassium at each visit. SZC was terminated for any drug-related adverse events, including hypokalemia (defined as serum potassium <3.5 mEq/L) or any other reasons at the discretion of the treating physicians. 

### 2.4. Potassium Intake Restriction

Patients with chronic hyperkalemia were educated and encouraged to consume <1500 mg/day of potassium according to our institutional protocol. Briefly, nutrition experts educated the patients on how to estimate and record their daily potassium consumption and how and why they should avoid potassium-rich foods. Following the initiation of SZC and the confirmation of improvement in hyperkalemia (i.e., serum potassium level <5.0 mEq/L), these mandated restrictions for potassium intake were liberated up to a daily potassium consumption <2000 mg under careful monitoring of dietary intake amount and serum potassium levels at each visit. The incremental dietary intake was confirmed by the attending clinicians.

### 2.5. Study Outcomes

The primary endpoint was the trend during the 3 month observational period in the various nutrition status scores: a controlling nutritional status (CONUT) score, a prognostic nutritional index (PNI) score, and a geriatric nutritional risk index (GNRI) score. These scores were calculated using serum albumin levels, serum total cholesterol levels, lymphocyte count, and body weight [5].

Secondary endpoints of interest were the trend in psoas major muscle volume, which was quantified by abdominal computed tomography [6], and the average days per one bowel movement, which was calculated from the numbers of bowel movement per month. 

### 2.6. Statistics

Statistics were conducted using SPSS Statistics 23 (SPSS Inc., Armonk, IL, USA). Two-tailed p-values <0.05 were considered statistically significant. All continuous variables were assumed to be non-parametric data considering the small sample size. Categorical variables were stated as numbers and percentages. Trends in clinical parameters were compared using the Wilcoxon signed-rank test.

## 3. Results

### 3.1. Baseline Characteristics

A total of 24 patients who continued SZC for at least 3 months were included. The median age was 78 (69, 83) years old, and 14 (58%) were men (Table 1). Body mass index was 21.2 (18.7, 23.2), and 15 (63%) had heart failure. All patients had hyperkalemia with a median serum potassium level of 5.4 (5.1, 5.9) mEq/L at baseline and received strict restriction of potassium intake beforehand. The median estimated glomerular filtration rate was 29.8 (15.0, 43.0) mL/min/1.73m^2^. Most of the patients (83%) received renin-angiotensin system inhibitors, and 63% received mineralocorticoid receptor antagonists. The baseline PNI score was 40.7 (35.9, 44.6), the baseline GNRI score was 95.5 (88.2, 100.4), and the baseline CONUT score was 5.0 (2.5, 5.5).

### 3.2. Trend in Serum Potassium Levels

Overall serum potassium level decreased significantly following 3 months of SZC therapy, from 5.4 (5.1, 5.9) to 4.4 (4.2, 4.9) mEq/L (*p* < 0.001; Table 2). Following the start of SZC, all patients were able to reduce their potassium intake. No patients had hypokalemia during the observational period. All patients who completed 3 months of SZC therapy were alive at the end of observation. 

### 3.3. Trends in Nutrition Status (Primary Endpoint)

**Figure 1:**
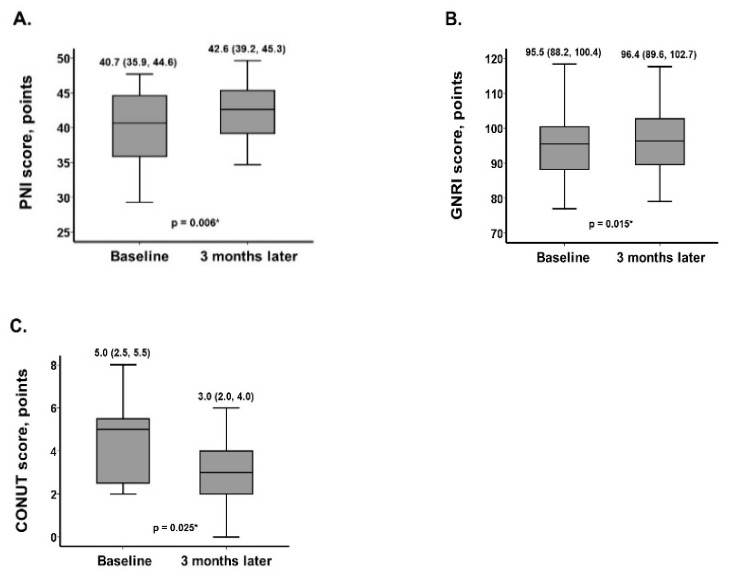
Trends of three nutrition scores during 3 months of SZC therapy, including PNI score (**A**), GNRI score (**B**), and CONUT score (**C**). * *p* < 0.05 by the Wilcoxon signed-rank test.

PNI score (from 40.7 [35.9, 44.6] points to 42.6 [39.2, 45.3] points) and GNRI score (from 95.5 [88.2, 100.4] points to 96.4 [89.6, 102.7] points) increased (i.e., improved) significantly at 3 months following the initiation of SZC therapy and liberation of potassium intake restrictions (*p* = 0.006 and *p* = 0.015, respectively; Figure 1a,b). CONUT score decreased (i.e., improved) significantly during the 3 month observational period from 5.0 (2.5, 5.5) points to 3.0 (2.0, 4.0) (*p* = 0.025; Figure 1c). 

### 3.4. Trends in other Clinical Parameters (Secondary Concerns)

**Figure 2:**
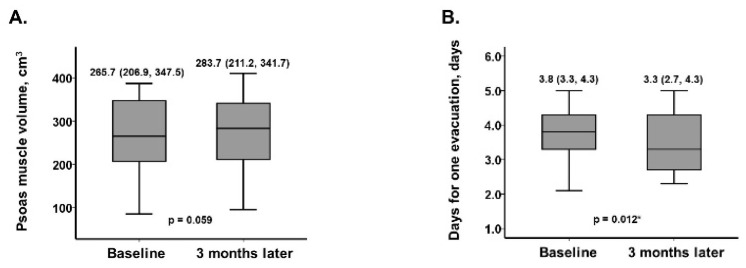
Trends of psoas muscle volume (**A**) and averaged days for one evacuation (**B**) during 3 months of SZC therapy. * *p* < 0.05 by the Wilcoxon signed-rank test.

Psoas muscle volume trended to increase from 265.7 (206.9, 347.5) cm^3^ to 283.7 (211.2, 341.7) cm^3^ (*p* = 0.059, N = 10, Figure 2a). Averaged days for one bowel movement decreased significantly from 3.8 (3.3, 4.3) days to 3.3 (2.7, 4.3) days (*p* = 0.012; Figure 2b). Trends in other laboratory data are displayed in Table 2. Serum albumin and lymphocyte count increased significantly (*p* = 0.025 and *p* = 0.040, respectively). Other variables remained unchanged (*p* >0.05 for all).

## 4. Discussion

In this study, we investigated nutrition status following 3 months of SZC therapy and purposeful liberation of potassium intake restriction in patients with hyperkalemia. Patients with hyperkalemia had some degree of malnutrition at baseline under strict potassium intake restriction, which was liberated in all patients following the initiation of SZC therapy. Following 3 months of SZC therapy, nutrition status, which was assessed using the PNI score, the GNRI score, and the CONUT score, improved significantly. 

### 4.1. Malnutrition in Patients with Hyperkalemia

As observed in our patients’ nutrition scores, many elderly patients with hyperkalemia had mildly or moderately poor nutrition status at baseline. Given our patients’ baseline characteristics, most of them had hyperkalemia, probably due to underlying chronic kidney disease and potassium-preserving neurohormonal blockers [7]. Such comorbidities may also independently affect nutrition status [5]. An indirect consequence of educating patients on potassium restriction may also affect the intake of other essential nutrients that improve nutrition status [3].

### 4.2. SZC Therapy and Loosening of Potassium Intake Restriction

SZC is a recently introduced novel potassium binder with robust evidence to normalize hyperkalemia and maintain serum potassium levels within a normal range with few drug-related adverse events, including hypokalemia [8,9]. Serum potassium levels remained within an appropriate range in our patients even after liberating potassium intake restriction. Of note, we did not quantify the actual amount of dietary intake.

### 4.3. Impact of SZC Therapy on Malnutrition

As hypothesized, all three nutrition scores, which are established scoring systems to assess nutrition status [1], improved following the initiation of SZC, improvement in hyperkalemia, and liberation of the potassium intake restriction. These findings were confirmed in the setting of improvement in psoas muscle volume [6].

A lower dietary potassium intake might be associated with a deficiency in muscle mass commonly seen in geriatric patients [10]. Strict restriction of potassium intake may also lower the intake of other high-quality nutrients and possibly contribute to lower muscle mass [11]. SZC therapy might indirectly assist patients with consuming an adequate diet by removing the need for a strict potassium-reduced diet by improving hyperkalemia and maintaining serum potassium levels within the normal range. A direct impact of SZC therapy on improving nutrition status remains uncertain.

Consistently, a systemic review demonstrated a protective effect of high dietary potassium intake on the progression of chronic kidney disease in six out of nine studies, whereas the other three reported neutral results [12]. Lower consumption of potassium-rich fruits and vegetables in the hemodialysis population was associated with higher all-cause and non-cardiovascular death [13]. In another study including hemodialysis patients, lower dietary potassium intake was associated with higher mortality [14].

These potassium-rich foods, including fibers, might have an advantage in preventing constipation [15,16]. SZC therapy and freedom from restriction of potassium intake might have another benefit in improving constipation. 

### 4.4. Limitations

This is a proof-of-concept study consisting of a small sample size, likely due to the limited prescription of SZC to treat hyperkalemia. We hope we can conduct larger-scale, multi-institutional studies to validate our findings following the incremental clinical use of SZC. We lack a control group in which hyperkalemia was managed without SZC. Such an intervention might not be ethically acceptable. Given the observational nature of this study, the causative mechanism of our findings is based on the hypothesis. We assumed that the liberation of potassium intake restriction resulted in the incremental intake of whole foods, but we did not accurately quantify the actual daily intake amounts. We do not deny the clinical implications of potassium intake restriction in an appropriate manner for some appropriate patients with good nutrition status. We used body weight data to calculate nutrition status based on the assumption that patients’ fluid status was relatively well-controlled on an out-patient basis using appropriate diuretics in all patients. In Japan, we cannot use other novel potassium binders like Patiromer.

## 5. Conclusions

Mid-term continuous SZC therapy and loosening of potassium intake restrictions might ameliorate malnutrition status in patients with chronic hyperkalemia.

## Figures and Tables

**Table 1:** Baseline characteristics.

	N = 24
Demographics	
Age, years	78 (69, 83)
Men	14 (58%)
Body mass index	21.2 (18.7, 23.2)
Comorbidity	
Diabetes mellitus	15 (63%)
Atrial fibrillation	9 (38%)
Ischemic heart disease	6 (25%)
Heart failure	15 (63%)
History of stroke	2 (8%)
Hemodialysis	3 (13%)
Medication	
Renin-angiotensin system inhibitors	20 (83%)
Mineralocorticoid receptor antagonists	15 (63%)

Continuous variables are displayed as medians (25% quartile, 75% quartile). Categorical variables are displayed as numbers and percentages.

**Table 2:** Trend in laboratory data during 3 months of SZC therapy.

	Baseline	Three Months Later	*p* Value
Serum potassium, mEq/L	5.4 (5.1, 5.9)	4.4 (4.2, 4.9)	<0.001 *
Serum albumin, g/dL	3.6 (3.3, 3.9)	3.7 (3.5, 4.0)	0.025 *
Hemoglobin, g/dL	11.3 (10.8, 12.1)	11.2 (10.2, 11.8)	0.057
Serum sodium, mEq/L	139 (136, 141)	140 (137, 141)	0.21
Total cholesterol, mg/dL	155 (135, 186)	160 (144, 179)	0.39
Lymphocyte, /μL	753 (501, 1088)	1044 (691, 1453)	0.040 *
eGFR, mL/min/1.73 m^2^	29.8 (15.0, 43.0)	30.4 (15.2, 45.6)	0.41

Continuous variables are displayed as medians (25% quartile, 75% quartile). eGFR or estimated glomerular filtration ratio. * *p* < 0.05 by the Wilcoxon signed-rank test.

## Data Availability

Data are available upon reasonable request from the corresponding author.
